# Quantitative evaluation of the results of digital forensic investigations: a review of progress

**DOI:** 10.1080/20961790.2020.1837429

**Published:** 2021-02-08

**Authors:** Richard E. Overill, Jan Collie

**Affiliations:** a Department of Informatics, King’s College London, London, UK; b Department of Computing & Communication, The Open University, Milton Keynes, UK

**Keywords:** Forensic sciences, digital forensic metrics, measures of plausibility, Bayesian networks, probability theory, statistical theory, complexity theory, information theory

## Abstract

Unlike conventional forensics, digital forensics does not at present generally quantify the results of its investigations. It is suggested that digital forensics should aim to catch up with other forensic disciplines by using Bayesian and other numerical methodologies to quantify its investigations’ results. Assessing the plausibility of alternative hypotheses (or propositions, or claims) which explain how recovered digital evidence came to exist on a device could assist both the prosecution and the defence sides in criminal proceedings: helping the prosecution to decide whether to proceed to trial and helping defence lawyers to advise a defendant how to plead. This paper reviews some numerical approaches to the goal of quantifying the relative weights of individual items of digital evidence and the plausibility of hypotheses based on that evidence. The potential advantages enabling the construction of cost-effective digital forensic triage schemas are also outlined.

**Key points**
- The absence of quantified results from digital forensic investigations, unlike those of conventional forensics, is highlighted.
- A number of approaches towards quantitative evaluation of the results of digital forensic investigations are reviewed.
- The significant potential benefits accruing from such approaches are discussed.

## Introduction

One of the most striking differences between the results reported from conventional forensic investigations, involving the examination of physical, chemical and biological material traces on the one hand, and those from digital forensic investigations on the other, is the absence of any quantitative measures of confidence, plausibility or uncertainty associated with the latter results. To illustrate, the random match probability (RMP) of two matching DNA profiles not belonging to the same person (or to identical twins) is ca.10^−8^, to within a factor of 10, depending on the number of loci in the profile and the size of population database [1]. Similarly, in forensic entomology examination of blow-fly larval instars can be used to determine the postmortem interval for a corpse, with a known range of uncertainty related to ambient temperature and humidity.

Such quantitative measures, generally derived the results of statistical analyses or laboratory experiments, are valuable since they enable both defence and prosecution sides to evaluate the strength (or weight) of an individual item of recovered evidence (e.g. a DNA profile) and, by extension, to estimate the strength (or plausibility) of a case built from many such evidential items.

This state of affairs is at least in part related to the relative maturity of conventional forensic science in comparison with digital forensics. We might tentatively trace the origin of systematic forensic science investigations to be ca.1900 with the publication of Edward Henry’s fundamental work on fingerprints [2], followed in 1901 by the establishment of the Fingerprint Branch at New Scotland Yard. The subsequent enunciation by Edmond Locard of his well-known Exchange Principle that every contact leaves a trace [3] led to important conceptual and methodological advances in the science. Similarly, Cliff Stoll’s tracking of the hacker Markus Hess [4] and Eugene Spafford’s decoding of the Robert Morris internet worm [5] could be taken as one measure of the beginning of systematic digital forensic investigations. It is immediately clear that conventional forensics has gained a head-start of around 90 years over digital forensics. However, given that digital forensic evidence is required to meet precisely the same admissibility criteria and tests of rigour in a court of law as conventional forensic evidence, it is apparent that there is a requirement to develop analogous quantifiable metrics for the findings of digital forensic investigations.

Fred Cohen, in particular, has made significant efforts to specify the rigorous scientific and engineering principles and practices upon which the requirements for such metrics should be based [6]. His work demonstrates that since individual binary bits have a physical instantiation it is possible to treat collections of them as a “bag of bits” using mathematical concepts from information physics, which can lead to quantitative findings. It is also worth noting that a recent Organization of Scientific Area Committees (OSAC) report [7] briefly considers the quantitative evaluation of investigative findings, and a Scientific Working Group on Digital Evidence (SWGDE) report [8] provides numerical error rates for some common digital forensic processes.

While there has been significant progress in specifying models and processes for the systematisation of digital forensic investigations, which are aimed at improving the consistency and reliability of the conclusions reached [9–15], it is important to emphasise that such developments, invaluable as they undoubtedly are within their own remit, do not attempt to directly address the issue of obtaining quantifiable findings from digital forensic investigations, analogous to those exemplified at the beginning of this section, which is the principal subject of this review article.

As a point of clarification, we should note here that the great majority of the work described in this review article refers to the quantification of hypotheses (propositions or claims) based on the digital evidence, rather than the quantification of the digital evidence itself; this is an important distinction. The only instances cited here involving the quantification of digital evidence itself occur in the examples where conditional probabilities (likelihoods) are assigned to the nodes of Bayesian networks based on surveys of experienced domain experts.

## Proposition plausibility metrics

The formal relationship between plausibility and probability can be conveniently expressed as follows: probabilities signify the quantities that define a particular monotonic scale on which degrees of plausibility can conveniently be measured [16].

Bayesian methods, based on the conditional probability theorem of Revd. Thomas Bayes in his renowned posthumously published essay [17], have recently been cited as one approach to gaining quantitative traction in conveying degrees of (un)certainty in digital forensic results [18]. For a hypothesis (or proposition, or claim) *H,* with a single mutually exclusive and exhaustive alternative *H̅,* and recovered evidence *E*, Bayes' Theorem can be conveniently expressed as:

$$\frac{\mathrm{Pr}(H|E)}{\mathrm{Pr}(\bar{H}|E)}=\frac{\mathrm{Pr}(H)}{\mathrm{Pr}(\bar{H})}.\frac{\mathrm{Pr}(E|H)}{\mathrm{Pr}(E|\bar{H})}$$

where the left-hand side quotient represents the posterior odds ratio, and on the right-hand side the first quotient represents the prior odds ratio while the second quotient represents the likelihood ratio (LR). This simple expression can be generalised in a straightforward manner to situations involving multiple mutually exclusive and exhaustive alternative hypotheses.

Judea Pearl showed how networks can be defined and constructed which permit the propagation of probabilities from initial priors to final posteriors, based on the values of intervening conditional probabilities [19,20]. One of the first attempts to apply such quantitative methods to the analysis of an actual digital forensic investigation was made by Chow and co-workers using a Bayesian network model of an illicit peer-to-peer (BitTorrent) uploading case from Hong Kong, China [21]. The prior probabilities were taken to be strictly noninformative and the requisite conditional probabilities (likelihoods) were elicited from a survey of 31 domain experts. This model yielded a posterior probability of ca.92.5% in favour of the hypothesis that an illicit upload had indeed occurred given that all 18 anticipated items of digital evidence were recovered. Although a credible alternative hypothesis was not available for this case against which to compare the result, it corresponds to an LR of ca.12.3 in favour of the prosecution hypothesis. In subsequent studies, the sensitivity of this result to the absence of one (ca.0.08%), two (2.0%) or more items of digital evidence was found to be consistently small, while its sensitivity to uncertainties in the conditional probability (likelihood) values populating the nodes of the Bayesian network was also inconsiderable at ca.0.25% [22].

From 20 typical cases of internet auction fraud prosecuted in Hong Kong, China, Bayesian networks for both the prosecution and the defence cases were created and the LR of these two alternative explanations for the existence of the recovered digital evidence was computed to be 164 000 in favour of the prosecution hypothesis [23]. This finding may be interpreted as providing “very strong support” for the prosecution’s hypothesis [24]. The conditional probabilities required for the Bayesian networks were sourced from a survey of the members of the Hong Kong Customs & Excise digital investigation team involved in the prosecutions. While LRs are generally regarded as the preferred way to present forensic findings when at least two mutually exclusive and exhaustive hypotheses are available, it should nevertheless also be mentioned that there has been considerable debate regarding the possible (mis)interpretation of LRs potentially resulting in misleading conclusions being drawn [25].

A third example of Bayesian network analysis involves an actual case from Hong Kong, China of a leaked confidential (Yahoo!) email; the prior probabilities were taken to be strictly noninformative and the conditional probabilities (likelihoods) were elicited by questioning a domain expert. With every anticipated item of digital evidence successfully recovered the posterior probability in favour of the prosecution hypothesis was ca.97.2%. While a credible alternative hypothesis was not available for this case against which to compare the result, it corresponds to an LR of ca.34.7 in favour of the hypothesis; however, both single-parameter and multi-parameter sensitivity analyses resulted in minimal perturbations to that value [26,27].

While Bayesian networks deal mainly with conditional probabilities, these quantities can on some occasions be difficult to obtain in a reliable manner. In such situations it may instead be possible to apply conventional (frequentist) probability theory to the evaluation of the plausibility of a hypothesis put forward by either the prosecution or the defence side. For example, in cases where a seized computer is found to contain a relatively small number of illicit images or videos (e.g. of child pornography) amongst a much larger number of non-illicit material (e.g. of adult pornography) it is possible to use an Urn Model [28,29] and the Binomial Theorem to calculate the probability of the inadvertent download defence, under the assumption of random browsing activity. In two actual cases from Hong Kong, China the 95% confidence interval for the plausibility of this defence was shown to be ca.[0.03%, 2.54%] and ca.[0.00%, 4.35%], respectively [30].

In cases where very large quantities of illicit materials are recovered from a seized computer, the Trojan horse defence (THD) [31,32] is sometimes invoked to explain their presence. In such situations an analysis of the complexity of the processes involved in the alternative hypothetical explanations can be instructive. Operational complexity models count the number of operations (e.g. at byte-level) required to achieve the presence of the recovered materials by each of the two alternative mechanisms; the principle of least contingency, which asserts that (*ceteris paribus*) a simpler mechanism is more likely than a more intricate mechanism, then enables the odds ratio for the two alternatives to be computed as the inverse of the ratio of their complexities. In a particular scenario involving the deposition of a single 1MB image the odds against the THD were calculated to be just 2.979:1; these odds were lengthened to 197.9:1 if an up-to-date malware scanner was known to be operational at the material time [33]. A similar complexity based analysis has also been used to compute the odds against the THD for the five most commonly occurring cyber-crimes in Hong Kong, China [34], where ratios of between 100:1 and 1 000:1 were found; these odds ratios were subsequently furnished with worst case uncertainty bounds using standard error propagation theory to investigate the possibility of overlapping lower and upper bounds between the criminal and the THD hypotheses, respectively [35].

Knowing the relative plausibility of alternative hypotheses explaining the existence of the recovered digital evidence can be a valuable tool for aiding the investigating authority in deciding whether or not to refer a case to the prosecuting authority, and equally in assisting the prosecution authority in deciding whether or not to proceed to trial. Conversely, such quantitative information could also be made use of by the defence side in deciding how to plead: if the prosecution’s case is overwhelmingly plausible then the defendant may be advised to plead guilty whereas if its plausibility is only marginal then a not guilty plea might be entered. In some jurisdictions it is also possible that an expert witness might be permitted to bring forward such data as part of their testimony at trial.

## Probative value metrics

The probative value (or strength, or weight) of an individual item of digital evidence in the context of a particular criminal case reflects the degree to which the presence of that item of evidence, if recovered, contributes to the overall plausibility of the hypothesis concerning the processes that created all of the recovered digital evidence. Perhaps the simplest method to achieve this is to take the difference in the posterior probabilities of the Bayesian network for the hypothesis in the presence and in the absence of that item of evidence [36]. A second method is to generate the so-called Tornado diagram for the Bayesian network, which shows the ordered range of variation in posterior probability due to each evidential item with respect to all the remaining items [37]. Another, still more sophisticated, approach is to use information theory: the Kullback–Leibler divergence of the Shannon entropy gives the information gain as a measure of the difference between the probability distributions for the Bayesian network with (*P*) and without (*Q*) that item of evidence:

$$D_{KL}(P\|Q)=\;\sum_{i}P_{i}\log\frac{P_{i}}{Q_{i}}$$

These three approaches lead to somewhat different orderings for the evidential weights of the BitTorrent case mentioned above [38].

In digital forensic investigations it is commonly the case that the individual items of digital evidence may, at least to a first approximation, be considered conditionally independent of one another. Knowing the relative importance of each individual item of digital evidence in an investigation can then enable an efficient digital investigation scheme to be devised in which the most highly probative evidential items are searched for first, in order, relegating the items of lesser importance until later. If one or more of the anticipated items of high importance are not recovered the search may be de-prioritised or even abandoned; conversely, if all of the anticipated items of high importance are found then it may not be considered necessary to search for those items of lowest importance as the overall plausibility of the investigative hypothesis would not be sensibly improved by doing so.

In addition, such priority-driven investigation schemes can be termed cost-effective if they are extended to utilise economic criteria such as Return on Investment (RoI) or Cost–Benefit Ratio (CBR), through a knowledge of the resources (e.g. personnel, time, specialised equipment, etc.) required to recover and analyse each anticipated item of digital evidence [39,40].

## Summary and conclusions

We have endeavoured to make the case that digital forensics should aim to catch up with conventional forensics in providing quantified results from its investigations, despite the intrinsically sensitive and volatile nature of much digital evidence. A number of methodologies, such as Bayesian networks, complexity theory, probability theory and statistics, and information theory, by which this may be accomplished have been outlined and some typical results have been summarised. The uses to which such quantitative results could be put in both investigative and juridical contexts have also been briefly explored. In particular it should be emphasised that by bringing digital forensics into line with conventional forensic science, expert witness testimony and examiners’ investigation reports can offer the courts a transparent rationale for the degree of certainty associated with their conclusions, rather than relying on previous relevant experience as the sole determinant of their expert opinion.

Ongoing and future lines of research in this area are likely to involve comparative studies of different measures of complexity [41] as applied to evaluating the plausibility of investigative hypotheses. Comparative studies of the divergence of various forms of entropy [42] applied to the quantification of evidential weight (or probative value) is also likely to be productive. Another potentially worthwhile avenue of research involves the pre-emptive analysis of the plausibility of novel, so-far-unused cybercriminal defences, for example the cosmic ray defence (CRD) where power law statistics guided by Moore’s law enabled the prediction of a 512-fold increase in the incidence of CR-induced memory bit-flips since 1994, unless protected by sufficiently powerful error correcting codes [43].
